# Fractal-Inspired Biorefineries for Sustainable Polymer Production: A Conceptual Framework Integrating Artificial Intelligence and Industry 5.0

**DOI:** 10.3390/polym18202505

**Published:** 2026-10-13

**Authors:** Young Chan Ko, Soon Wan Kweon, Yong Ju Lee, Byoung Geun Moon, Hyoung Jin Kim

**Affiliations:** 1 YK Consulting Co., Ltd., 17 Apkujung-ro 11-gil, Kangnam-gu, Seoul 06000, Republic of Korea; youngko2004@hanmail.net; 2 Separation & Purification Research Center, Chemical & Process Technology Division, Korea Research Institute of Chemical Technology, 141 Gajeong-ro, Yuseong-gu, Daejeon 34114, Republic of Korea; dhks615@kookmin.ac.kr; 3 Department of Forest Products and Biotechnology, Kookmin University, 77 Jeongneung-ro, Seongbuk-gu, Seoul 02707, Republic of Korea; 4 Consumer Product Division Packaging Technology Center, Korea Conformity Laboratories, 199 Gasan Digital 1-ro, Geumcheon-gu, Seoul 08503, Republic of Korea; bioman1994@kcl.re.kr

**Keywords:** fractal-inspired design, biorefinery, lignocellulosic biomass, sustainable polymers, artificial intelligence, Industry 5.0, process integration, biomass-to-chemicals

## Abstract

The transition from petroleum-derived polymers to renewable materials requires biorefineries capable of managing the compositional variability and multiscale structure of lignocellulosic biomass. This conceptual narrative review examines fractal-inspired principles—including hierarchical organization, branching, modularity, and finite-range statistical self-similarity—as a design perspective for integrated biorefineries. Deterministic mathematical fractals are distinguished from statistical fractals and hierarchical engineered systems, and methods for characterizing scale-dependent surfaces, porous catalysts, and transport networks are reviewed. The coordinated valorization of cellulose, hemicellulose, and lignin is then considered within an integrated biomass-to-chemicals framework for producing platform chemicals, functional materials, and polymer precursors. Petroleum crude-to-chemicals processing, including the S-OIL Shaheen Project, is discussed as a limited industrial benchmark for integrated fractionation, residue upgrading, and product diversification rather than as direct evidence of fractal engineering. An integrated framework is proposed in which fractal-inspired design provides the physical and organizational architecture, artificial intelligence supports characterization, prediction, optimization, and control, and Industry 5.0 provides the objectives of sustainability, resilience, and human-centered manufacturing. The framework remains conceptual. Its potential benefits must be evaluated through transport modeling, mass and energy balances, process simulation, experimental comparison, techno-economic analysis, and life-cycle assessment.

## 1. Introduction

The transition from fossil resources to renewable carbon is creating a need for integrated biorefineries that can produce chemicals, materials, and polymer precursors from lignocellulosic biomass. Although biomass is renewable, its conversion is complicated by variations in composition, moisture, ash, structure, and supply. These variations influence fractionation, diffusion, reaction, separation, product quality, and overall process economics [1,2].

Existing biorefinery research has developed through several complementary frameworks, including whole-biomass valorization, lignin-first processing, process intensification, hierarchical catalyst design, multiscale process engineering, and the circular bioeconomy. These approaches seek to improve the utilization of cellulose, hemicellulose, lignin, and other biomass components while reducing energy consumption, waste generation, and carbon losses [2,3,4].

Fractal and hierarchical structures occur in many natural and engineered systems. Branching networks distribute fluids in trees and biological tissues, while multilevel pore structures influence transport in catalysts, membranes, and porous polymers. Such examples suggest that hierarchy, branching, and multiscale connectivity may provide useful design principles for complex biorefinery systems. However, not every hierarchical structure is mathematically fractal, and visual resemblance alone does not demonstrate self-similarity, scale invariance, or superior engineering performance [5,6,7].

Artificial intelligence provides a separate but complementary capability. Machine learning can assist biomass characterization, catalyst screening, product-yield prediction, process optimization, control, anomaly detection, and predictive maintenance. Industry 5.0 establishes the broader objectives within which these technologies should operate: sustainability, resilience, and human-centered manufacturing [8,9].

Research on fractal engineering, AI-assisted processing, integrated biorefineries, and Industry 5.0 has largely developed in separate fields. A critical synthesis is therefore needed to determine how these concepts are related, where the fractal terminology is justified, and how any proposed engineering advantage should be evaluated.

This article presents a conceptual narrative review. It proposes an integrated framework in which fractal-inspired design provides the physical and organizational architecture, AI provides analytical and operational intelligence, and Industry 5.0 defines the system objectives. The framework is not presented as an experimentally validated process design. Instead, it identifies testable relationships, quantitative performance criteria, and research priorities for sustainable polymer production.

This review addresses three questions:

- Which fractal or hierarchical principles are relevant to biorefinery materials, reactors, and process networks?
- How can AI support the design and operation of these multiscale systems?
- Which quantitative methods are required to determine whether the proposed framework improves technical, economic, and environmental performance?

This article is a conceptual narrative review rather than a systematic review, meta-analysis, or detailed process-design study. The literature is organized into five connected themes: fractal and hierarchical engineering, lignocellulosic biomass valorization, petroleum and biomass refinery integration, AI-assisted process engineering, and Industry 5.0.

The review distinguishes three types of evidence. First, established evidence describes measured structures, conversion pathways, and engineering performance reported in the literature. Second, conceptual interpretation identifies possible relationships among hierarchy, multiscale organization, and integrated processing. Third, proposed research directions identify hypotheses that require experimental, computational, economic, or environmental validation.

## 2. Fractal and Hierarchical Engineering Principles

### 2.1. Deterministic Mathematical Fractals

To illustrate the fundamental concepts of fractal geometry, four classical deterministic fractals—Cantor set, Koch curve, Sierpiński carpet, and Menger sponge—are briefly introduced.

Although generated by simple recursive rules, these structures illustrate self-similarity, recursion, scale-dependent geometry, and the emergence of complexity through repeated iteration [5,10,11].

**Figure 1:**
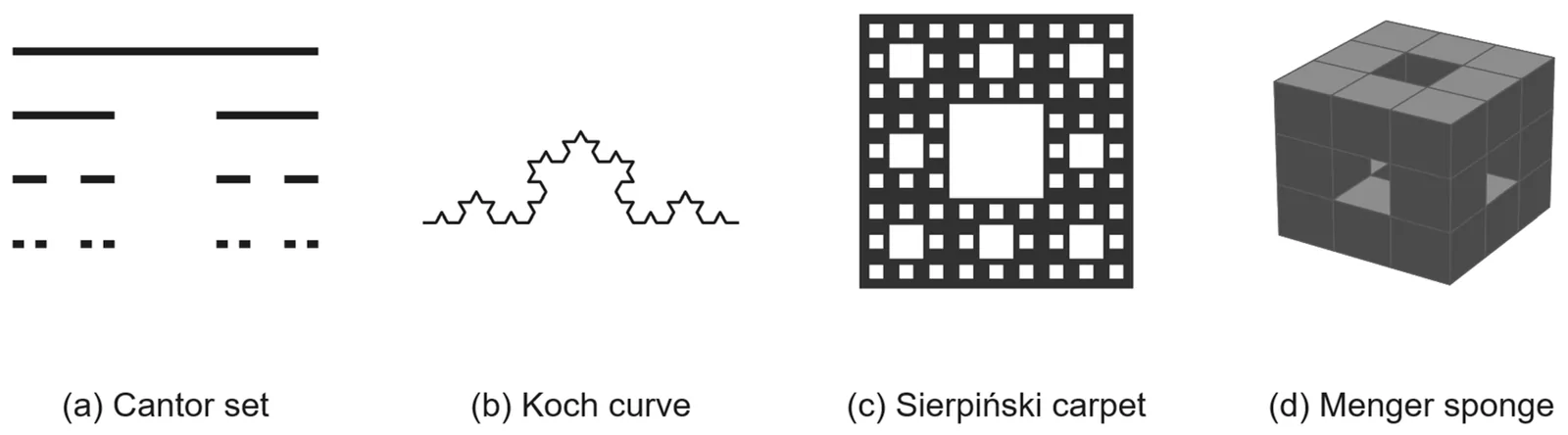
Representative deterministic fractals used to illustrate recursive self-similarity: (**a**) Cantor set; (**b**) Koch curve; (**c**) Sierpiński carpet; and (**d**) Menger sponge.

Lower-dimensional deterministic fractals, such as the Koch curve, illustrate how recursive construction can increase geometric complexity or boundary length, but their extension to three-dimensional physical systems is not direct. In real porous materials, catalysts, and transport networks, mass transfer depends not only on geometric complexity or interfacial area but also on pore connectivity, tortuosity, diffusion length, and accessibility. Thus, increasing geometric complexity does not necessarily improve mass-transfer performance, and the engineering significance of a fractal-inspired structure must be evaluated independently through appropriate three-dimensional structural and transport measurements. These representative deterministic constructions are illustrated in Figure 1.

Table 1 summarizes the mathematical structures, design analogies, and limitations of these idealized fractals. They are used here as conceptual models rather than as direct representations of real catalysts or biorefinery equipment.

### 2.2. Statistical Fractals and Hierarchical Systems

Real biomass structures, catalyst surfaces, porous polymers, and transport networks are generally not exact deterministic fractals. Some exhibit statistical self-similarity over a finite range of scales, whereas others are hierarchical structures for which fractal scaling has not been demonstrated. The terms fractal, statistically fractal, fractal-inspired, and hierarchical must therefore be distinguished carefully [5,11].

In this review, these terms are used as follows:

Fractal: A structure whose scaling characteristics are mathematically defined or quantitatively established over a specified range of scales.

Statistically fractal: A structure exhibiting approximate statistical self-similarity or power-law scaling over a finite range of scales.

Fractal-inspired: An engineered structure or system deliberately designed using principles associated with fractals, such as branching, recursion, self-similarity, or multiscale organization, without necessarily satisfying strict mathematical criteria for fractality.

Hierarchical: A structure or system organized at multiple levels or scales but without demonstrated fractal scaling.

**Figure 2:**
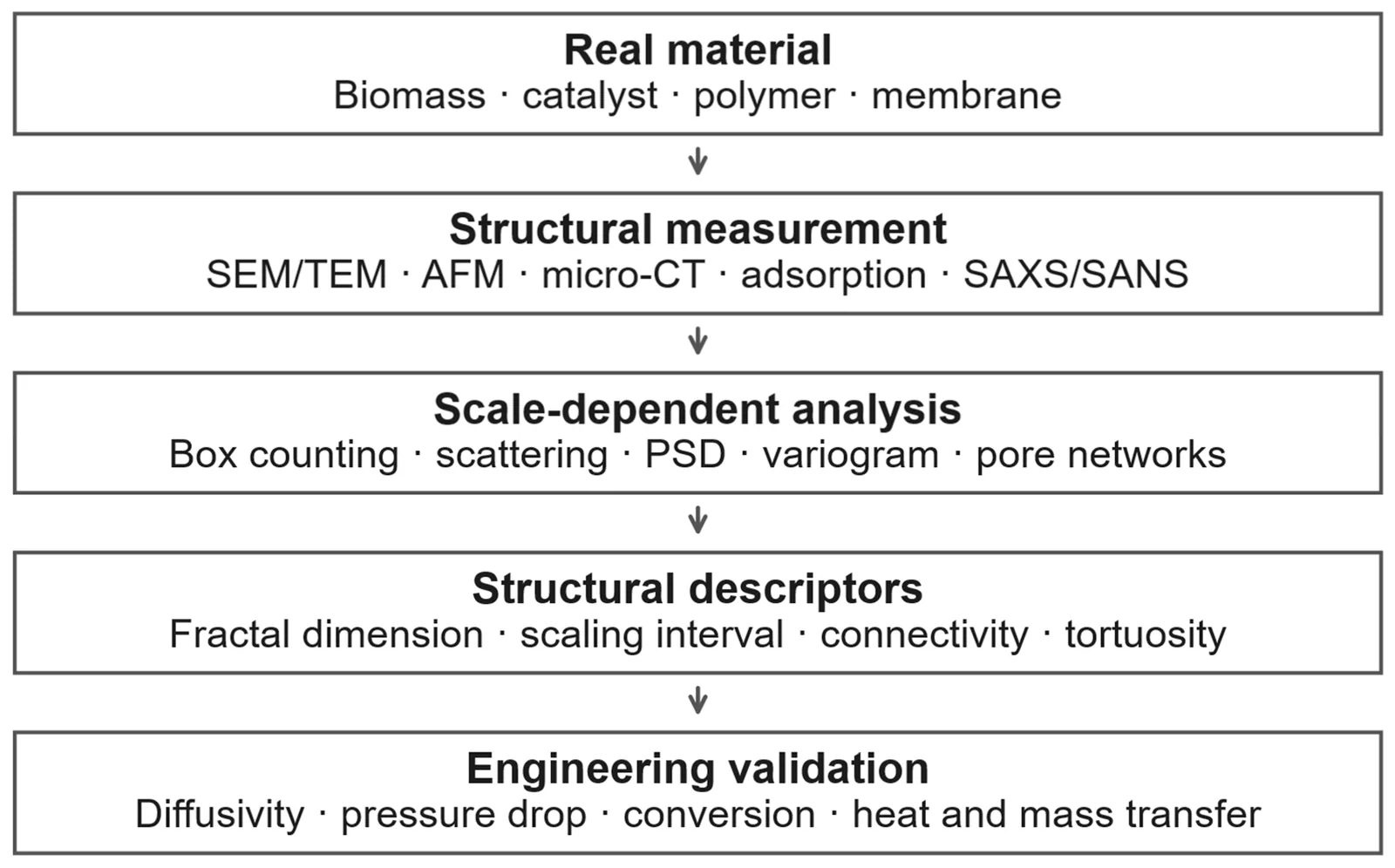
Experimental characterization and engineering validation of fractal and hierarchical structures.

Figure 2 summarizes the experimental characterization and engineering validation of fractal and hierarchical materials. A real material—such as biomass, a porous catalyst, a polymer network, or a membrane—is first characterized using structural measurement techniques, including scanning electron microscopy (SEM), transmission electron microscopy (TEM), atomic force microscopy (AFM), X-ray microcomputed tomography (micro-CT), gas adsorption, small-angle X-ray scattering (SAXS), or small-angle neutron scattering (SANS). The resulting data may then be analyzed using box-counting, scattering power laws, power spectral density (PSD), variograms, or pore-network analysis.

These analyses provide structural descriptors such as fractal dimension, scaling range, pore-size distribution, connectivity, and tortuosity. Their engineering significance must subsequently be determined through independent measurements of diffusivity, permeability, pressure drop, conversion, selectivity, and heat- and mass-transfer performance.

The box-counting method is described by Equation (1):

(1) $$\log N\left(\varepsilon \right)=D\log\left(1/\varepsilon \right)+C$$

where *N*(*ε*) is the number of boxes of side length ε required to cover the structure, *D* is the box-counting dimension estimated from the slope of the log–log plot, and *C* is a constant. Linear regression should be applied only over a clearly identified finite scaling region. Real engineering materials generally do not exhibit perfect scale invariance across all length scales.

Thus, fractal dimension describes scale-dependent structural complexity; engineering performance must be validated independently.

### 2.3. Fractal Characterization

For an ideal self-similar fractal, the similarity dimension is given by Equation (2) [11]:

(2) $$D=\frac{\log N}{\log\left(1/r\right)}$$

where *N* is the number of self-similar elements at each iteration and *r* is their linear scale relative to the parent structure.

For example, the Sierpiński triangle produces three copies at one-half the parent scale, giving *D* = log(3)/log(2) = 1.585. This relationship is appropriate for ideal deterministic constructions; real biomass, polymers, catalyst surfaces, and porous materials generally require experimental assessment over a defined range of length scales.

Equation (2) applies to ideal deterministic fractals, such as the Cantor set, Sierpiński triangle, Sierpiński carpet, and Menger sponge. Real biomass, polymers, catalyst surfaces, and porous materials, however, rarely possess such exact self-similarity. Their fractal characteristics must therefore be measured experimentally over a defined range of length scales.

Other techniques characterize fractality without relying solely on images. Gas adsorption can be used with surface-fractal models to characterize complex pore surfaces. Small-angle X-ray scattering (SAXS) and small-angle neutron scattering (SANS) can reveal mass- or surface-fractal scaling through power-law scattering behavior. Power spectral density (PSD) analysis evaluates scale-dependent surface roughness in the frequency domain, while variogram analysis examines how spatial variation changes with separation distance. Pore-network reconstruction, particularly from three-dimensional imaging, can additionally quantify connectivity, branching, pore-size distributions, and transport pathways.

The important point is that these methods do not necessarily measure the same fractal dimension. A box-counting dimension obtained from a two-dimensional SEM image, for example, should not automatically be treated as equivalent to a surface-fractal dimension obtained from adsorption or SAXS. Each dimension has a particular mathematical and physical meaning.

Every reported fractal dimension should therefore specify the measurement method, spatial resolution, scale interval, fitting procedure, regression quality, uncertainty, and physical interpretation. These requirements answer seven different questions: How was the structure measured? What was the smallest feature that could be resolved? Over what range was scaling actually observed? How was the dimension calculated? How well did the scaling law fit the data? How reliable is the resulting value? And what does that dimension physically represent?

The scale interval is especially important. A straight line on a log–log plot does not by itself prove that an entire material is fractal. If power-law behavior exists only from, for example, 1 to 20 μm, the appropriate conclusion is that the material exhibits statistical fractal scaling over that finite range, not that it is universally self-similar.

Accordingly, a material is described as statistically fractal in this paper only when scale-dependent measurements demonstrate a reproducible scaling relationship over a defined range of length scales. Otherwise, the terms hierarchical or fractal-inspired are used.

### 2.4. Fractal Dimension and Engineering Performance

Fractal dimension is a structural descriptor rather than a direct performance index. A higher fractal dimension may indicate greater boundary complexity, roughness, or space-filling capacity within a defined scale interval; however, it does not necessarily correspond to a larger accessible surface area, lower transport resistance, or improved catalytic performance. Engineering performance also depends on other structural and operating parameters, including pore connectivity, tortuosity, pore constrictions, reaction kinetics, fluid properties, pressure drop, fouling, and operating conditions.

Accordingly, fractal characterization should be combined with system-specific engineering measurements when evaluating the performance of catalysts, membranes, fluid-distribution networks, heat exchangers, and porous polymers. Representative relationships between structural descriptors and engineering performance metrics are summarized in Table 2.

Thus, fractal dimension should be used to characterize structural complexity, while engineering performance should be validated independently using application-specific transport, reaction, mechanical, or thermal metrics.

This distinction is important. For example, a catalyst with a higher surface fractal dimension could have a more geometrically complex surface but poorer effective diffusion if narrow pore throats or high tortuosity restrict molecular transport. Likewise, a highly branched fluid manifold may distribute fluid more uniformly but could impose an unacceptable pressure-drop penalty. Therefore, “higher fractal dimension = better performance” should be avoided throughout the manuscript.

## 3. Integrated Valorization of Lignocellulosic Biomass

Lignocellulosic biomass is an abundant renewable feedstock with potential for the production of fuels, chemicals, fibers, materials, and polymer precursors. Although some fiber and material applications are established, the technical and economic feasibility of many integrated fuel, chemical, and polymer-precursor pathways continues to be assessed. Unlike petroleum, however, biomass is chemically and structurally heterogeneous, and its composition varies with plant species, tissue type, geographic origin, maturity, growing conditions, harvesting season, storage, and processing history. Efficient biorefinery operation therefore requires both selective fractionation of the major biomass components and sufficient process flexibility to accommodate feedstock variability [1,2].

From an integrated biorefinery perspective, lignocellulosic biomass should not be regarded simply as a source of energy or a single product. Rather, its major components—cellulose, hemicellulose, and lignin—represent complementary feedstocks that can be separated and converted through different pathways into multiple value-added products. This concept provides the basis for the Biomass-to-Chemicals (B2C) framework discussed in subsequent sections.

**Figure 3:**
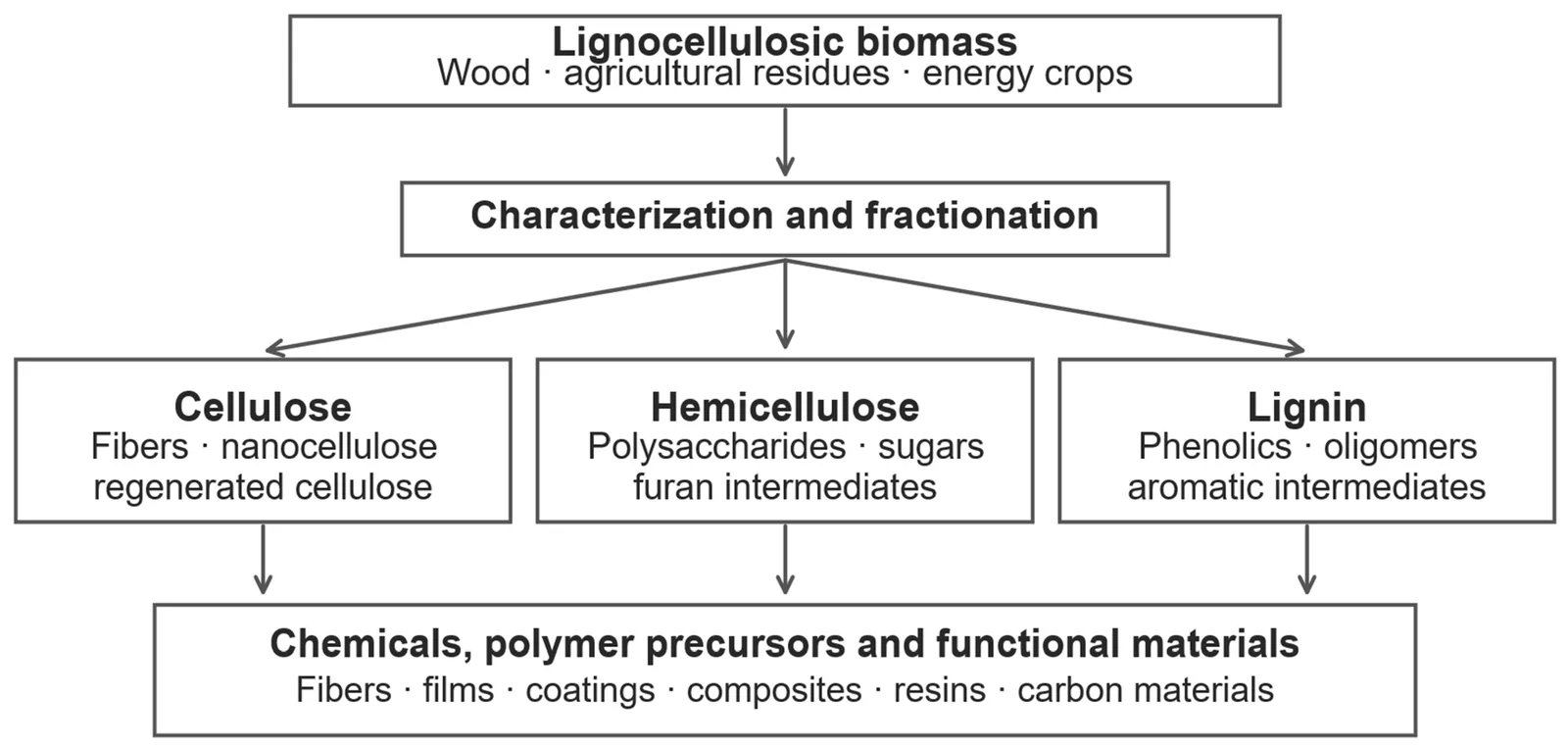
Integrated valorization pathways for lignocellulosic biomass.

Figure 3 illustrates the integrated valorization pathways for lignocellulosic biomass. Lignocellulosic feedstocks are first characterized and fractionated into complementary cellulose-, hemicellulose-, and lignin-rich streams. Each fraction is then directed toward conversion pathways suited to its molecular structure. Cellulose can be converted into fibers, nanocellulose, regenerated cellulose, fermentable sugars, and carbohydrate-derived intermediates [12,13,14,15]. Hemicellulose can provide functional polysaccharides, C5 and C6 sugars, and furan-based intermediates [16,17]. Lignin can be converted into phenolic fractions, functionalized oligomers, aromatic intermediates, and carbon-rich materials [4,18,19,20,21]. The coordinated valorization of these three fractions can create a hierarchical product network for the integrated production of chemicals, polymer precursors, and functional materials.

Representative experimental demonstrations include the mechanical fibrillation of pulp fibers into cellulose nanofibers by Iwamoto et al. [22], the production of soluble sugars from cellulose and hemicellulose in lignocellulosic feedstocks by Luterbacher et al. [23], and the recovery of lignin-derived phenolic monomers, dimers, and oligomers by Van den Bosch et al. [24]. These studies support the individual pathways summarized in Figure 3 but do not establish a single commercially validated process encompassing all listed products.

In the figure, the arrows indicate the branching pathways representing hierarchical fractionation and product diversification; they do not, by themselves, demonstrate mathematical self-similarity or scale invariance.

### 3.1. Biomass Composition and Variability

Lignocellulosic biomass consists primarily of cellulose, hemicellulose, and lignin, together with smaller quantities of extractives, inorganic constituents, and water. The relative abundance, molecular structure, and spatial organization of these components strongly influence pretreatment requirements, fractionation efficiency, catalytic conversion, and final product distribution [12,13].

Cellulose is a linear polysaccharide composed of β-(1 → 4)-linked glucose units organized into crystalline and less ordered regions. Extensive intermolecular and intramolecular hydrogen bonding contributes to the strength and chemical resistance of cellulose. Depending on the fractionation and conversion route, cellulose can be utilized for pulp and fibers, nanocellulose, regenerated cellulose, fermentable glucose, biofuels, and bio-based materials.

Hemicellulose comprises a heterogeneous group of branched polysaccharides, including xylans, mannans, and related carbohydrates. Compared with cellulose, hemicellulose generally has a lower degree of polymerization and is more readily hydrolyzed. It can therefore serve as a source of fermentable sugars and platform chemicals, including furfural from pentose-rich fractions and xylitol from xylose.

Lignin is a heterogeneous aromatic biopolymer associated with the carbohydrate components of the plant cell wall. It contributes mechanical rigidity, hydrophobicity, and resistance to biological degradation. Because lignin is a major renewable source of aromatic carbon, its valorization is particularly important for the production of phenolic compounds, aromatic chemicals, carbon materials, adhesives, and bio-based polymer precursors [3,25].

Extractives include lower-molecular-weight compounds such as fatty acids, waxes, terpenes, sterols, and phenolic compounds. Although present in relatively small quantities, extractives can influence pulping, fractionation, fermentation, and catalytic processes and may also provide valuable specialty chemicals. Ash, representing the inorganic fraction, contains minerals such as potassium, calcium, magnesium, and silica. High ash concentrations may contribute to fouling, slagging, corrosion, and catalyst deactivation.

Moisture content affects biomass storage, transportation, drying requirements, and conversion efficiency. Its importance is particularly pronounced in thermochemical processes, where excessive moisture increases energy demand and may reduce overall process efficiency.

The composition of biomass is not constant. Even within a single species, chemical composition and physical structure may vary with plant age, tissue, season, climate, soil conditions, and storage history. Consequently, compositional values should generally be reported as representative ranges rather than universal constants. Feedstock variability should therefore be incorporated into biorefinery design through appropriate characterization, pretreatment, process control, and flexible product-routing strategies.

### 3.2. Representative Lignocellulosic Resources

A wide range of agricultural and forestry resources can provide lignocellulosic feedstocks for integrated biorefineries. Rather than treating each resource as an independent conversion system, representative feedstocks can be compared according to their structural characteristics, major components, and potential valorization pathways (Table 3).

Kenaf, sugarcane bagasse, and oil-palm residues illustrate how geographically and structurally different biomass resources can enter similar fractionation and valorization pathways. The objective of an integrated biorefinery is therefore not to develop an entirely separate process architecture for every feedstock, but to establish sufficiently flexible fractionation and conversion platforms capable of accommodating differences in composition and structure [26,27,28].

Black liquor represents a different but strategically important case. During kraft pulping, a substantial fraction of lignin and other organic constituents is transferred from wood into black liquor. Conventionally, these dissolved organics are concentrated and combusted in the recovery boiler to generate energy while recovering inorganic pulping chemicals. Partial recovery and upgrading of lignin, however, provides an opportunity to divert a portion of this renewable aromatic carbon toward higher-value chemicals and materials while maintaining the essential chemical- and energy-recovery functions of the kraft mill [3].

### 3.3. Hierarchical Fractionation and Component Valorization

Integrated biomass valorization begins with the selective separation of lignocellulose into major component streams. Pretreatment and fractionation processes disrupt the complex association among cellulose, hemicellulose, and lignin, allowing each fraction to be directed toward conversion pathways suited to its chemical characteristics.

The resulting product architecture can be represented as a hierarchical network:

Biomass → fractionation → cellulose/hemicellulose/lignin → intermediates → chemicals and materials.

Cellulose may be processed directly into fibers, nanocellulose, or regenerated cellulose, or hydrolyzed to glucose for subsequent biological or catalytic conversion. Hemicellulose can provide C5 and C6 sugars and associated platform chemicals, particularly furfural and xylitol from pentose-rich streams. Lignin can be recovered and subjected to depolymerization, hydrogenolysis, hydrodeoxygenation, pyrolysis, or other conversion processes to produce phenolics, aromatic intermediates, carbon materials, and polymer precursors.

This hierarchical product network is fractal-inspired rather than necessarily fractal in the mathematical sense. Its relevance lies in repeated branching and multilevel organization: a single renewable resource is separated into several primary fractions, which are subsequently converted into increasingly differentiated product streams. The architecture therefore provides a useful conceptual framework for integrated resource utilization without implying that the overall biorefinery possesses a measurable fractal dimension.

### 3.4. From Feedstock Variability to Integrated Valorization

The principal challenge in lignocellulosic biorefineries is not simply the separation of biomass components but the coordinated utilization of all major fractions. Maximizing one product while discarding or inefficiently utilizing the remaining fractions can compromise the economic and environmental advantages of the overall process.

An integrated strategy therefore seeks to match each biomass component with an appropriate conversion pathway while considering product value, energy consumption, chemical recovery, catalyst requirements, waste generation, and process integration. The optimum pathway may differ among kenaf, bagasse, oil-palm residues, wood, and other biomass resources because of differences in composition and structure.

Advanced sensing, process analytical technologies, and AI-assisted optimization may further improve this flexibility by characterizing incoming feedstocks, predicting processing behavior, and adjusting operating conditions or product routing. In this way, feedstock variability can be treated as a design and optimization variable rather than solely as a processing limitation.

The integrated utilization of cellulose, hemicellulose, lignin, and associated components establishes the material foundation for the Biomass-to-Chemicals (B2C) concept developed in the following sections. Under this framework, hierarchical fractionation, component-specific conversion, and integrated process optimization are combined to maximize the value obtained from renewable biomass while minimizing waste and resource consumption.

## 4. Lessons from Petroleum Refining

Petroleum refining provides a useful industrial benchmark for integrated resource conversion. Modern refineries combine feedstock separation, catalytic conversion, product purification, heat and energy integration, residue upgrading, and plant-wide optimization to convert a heterogeneous carbon feedstock into a broad portfolio of fuels, chemicals, and material precursors. These principles have been developed over decades to improve resource utilization, process efficiency, and product value [29,30].

The relevance of petroleum refining to biorefineries lies primarily in these system-level principles, rather than in direct similarities between petroleum and biomass chemistry. Lignocellulosic biomass differs fundamentally from crude oil in composition, molecular structure, processing requirements, and supply characteristics. Therefore, petroleum refining should be considered an engineering benchmark from which selected principles can be adapted, rather than a direct technological model for biomass conversion.

### 4.1. Transferable Refinery Principles

Several principles of petroleum refining are relevant to the development of integrated lignocellulosic biorefineries.

Integrated feedstock fractionation: Petroleum refining begins by separating crude oil into fractions with different physical and chemical properties. Although biomass cannot be separated by conventional distillation in an analogous manner, the general principle of dividing a heterogeneous feedstock into useful fractions is transferable. In a biorefinery, pretreatment and fractionation separate or selectively recover cellulose, hemicellulose, lignin, extractives, and other components for subsequent conversion.

Conversion and separation: Petroleum refineries integrate catalytic cracking, hydrocracking, reforming, hydrotreating, and separation processes to convert intermediate fractions into higher-value products. Similarly, biorefineries require coordinated chemical, catalytic, thermochemical, and biological conversion processes followed by efficient product separation and purification. Integration of conversion and separation is therefore important for both systems.

Residue upgrading: Modern petroleum refineries seek to minimize the production of low-value heavy residues by converting them into fuels and chemical feedstocks. This principle is particularly relevant to lignocellulosic biorefineries, where lignin and other difficult-to-convert streams have traditionally been used primarily for process energy. Higher-value utilization of lignin through catalytic depolymerization, functionalization, carbon-material production, and polymer applications can increase the overall value obtained from biomass [3,4,25].

Heat and energy integration: Petroleum refineries extensively recover and redistribute heat among process units to reduce external energy requirements. Similar integration is essential in biorefineries because pretreatment, fractionation, evaporation, drying, reaction, and separation can be energy intensive. Heat recovery and appropriate utilization of process residues can improve overall energy efficiency.

Product diversification: Petroleum refineries produce multiple products from a common feedstock rather than relying on a single product stream. The same principle is fundamental to the biorefinery concept. Cellulose, hemicellulose, lignin, and extractives can be directed toward different product families, including fibers, sugars, fuels, platform chemicals, aromatic compounds, carbon materials, and polymer precursors [2].

Plant-wide optimization: The performance of an integrated refinery cannot be optimized by maximizing each individual unit operation independently. Feedstock characteristics, energy demand, product yields, recycle streams, catalyst performance, environmental constraints, and market requirements interact across the entire facility. Future biorefineries likewise require plant-wide optimization rather than isolated optimization of individual conversion processes. Advanced process control, digital technologies, and AI-assisted optimization may further facilitate dynamic allocation of variable biomass resources among alternative product pathways.

These transferable principles can be summarized as:

Feedstock → fractionation → conversion → separation → upgrading → diversified products.

The same general architecture can guide biomass valorization, although the chemistry and unit operations required are fundamentally different.

### 4.2. Shaheen as a Limited Industrial Benchmark

The S-OIL Shaheen Project in Ulsan, South Korea, provides a contemporary example of closer integration between petroleum refining and petrochemical production. S-OIL states that the project includes a large steam cracker, polymer facilities, and the world’s first commercial deployment of Saudi Aramco’s Thermal Crude-to-Chemicals (TC2C™) technology, which converts crude oil and refinery by-products into petrochemical feedstocks [31].

Shaheen is relevant to the present discussion primarily as an industrial integration and product-diversification benchmark. Its significance lies in the coordinated use of refinery streams, conversion technologies, and downstream polymer production within an integrated processing system rather than in any demonstrated fractal architecture [31]. Because the project has not yet entered commercial operation and detailed performance data for the proprietary TC2C technology are not publicly available in the source used here, quantitative comparison of conversion efficiency, product yields, or operating performance with biomass-based processes would be premature. The benchmark is therefore limited to the publicly described process configuration and its strategy of integrating feedstock conversion with diversified downstream petrochemical and polymer production.

Shaheen is not a biorefinery, a demonstrated fractal system, or experimental validation of the fractal-inspired framework proposed in this paper. Its relevance is limited to the engineering philosophy of process integration, feedstock utilization, product diversification, and value enhancement. Consequently, the proposed Biomass-to-Chemicals (B2C) framework should be regarded as a conceptual extension of selected integration principles rather than as a biomass analogue of the specific Shaheen process technology.

### 4.3. Limits of the Petroleum–Biomass Analogy

Petroleum refineries and biorefineries share the general objective of converting complex carbon feedstocks into diversified portfolios of fuels, chemicals, materials, and polymer precursors. Both employ successive stages of feedstock separation, conversion, product recovery, residue upgrading, and energy integration. These organizational similarities make petroleum refining a useful industrial reference for integrated value creation, but they do not establish chemical or technological equivalence between petroleum and biomass processing.

**Figure 4:**
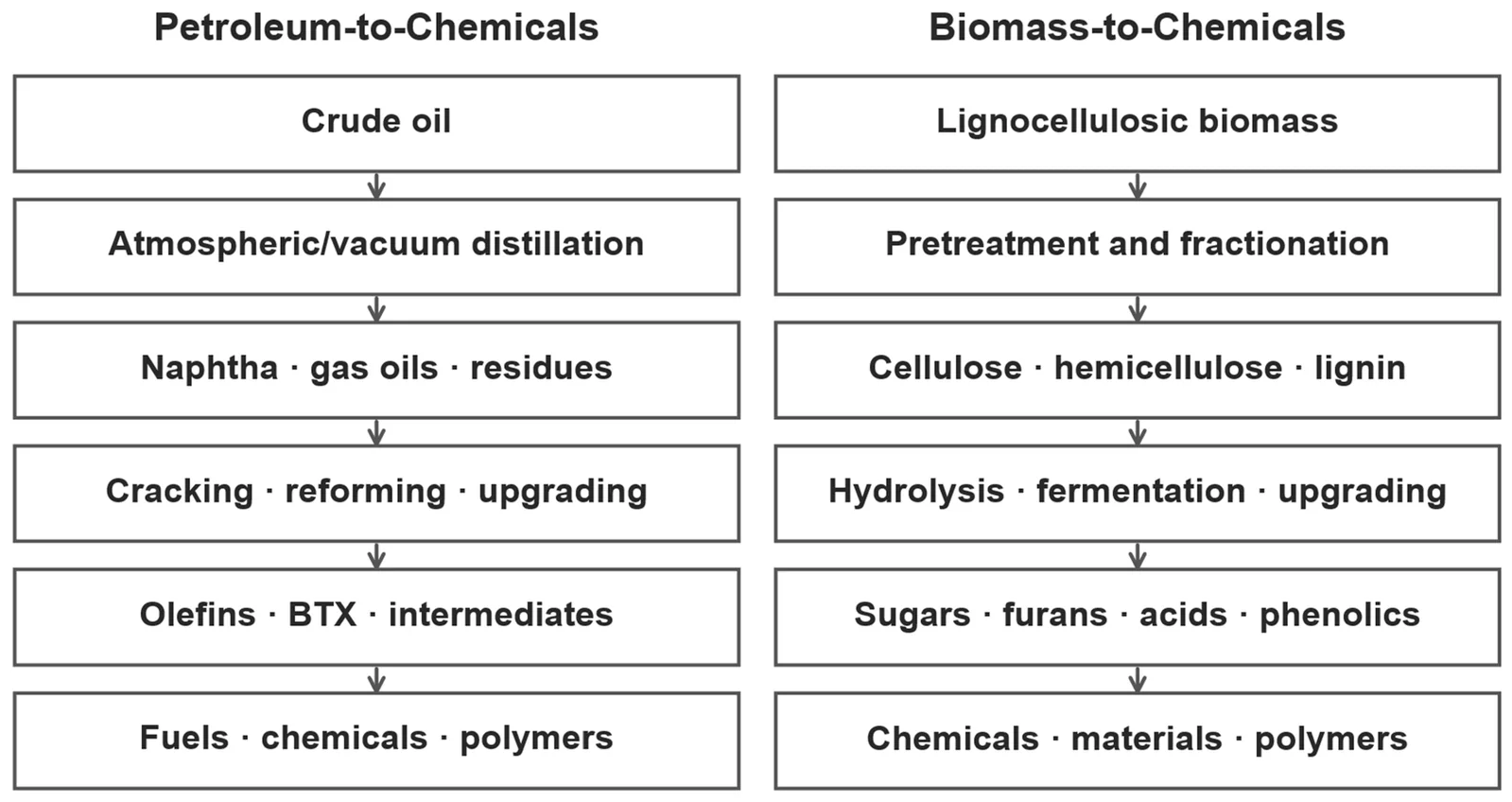
Conceptual comparison of petroleum-to-chemicals and biomass-to-chemicals pathways.

Figure 4 compares the principal pathways of petroleum-to-chemicals and biomass-to-chemicals processing. The comparison is conceptual and systems-oriented rather than chemical or quantitative.

Petroleum consists primarily of hydrocarbons and is initially separated according to volatility through atmospheric and vacuum distillation. Subsequent processes—including cracking, reforming, hydrocracking, and hydrotreating—modify molecular size, structure, and hydrogen content to produce fuels, olefins, aromatics, and other chemical intermediates [29,30].

Lignocellulosic biomass, by contrast, is an oxygen-rich and structurally heterogeneous material composed principally of cellulose, hemicellulose, and lignin. Its processing usually begins with physical, chemical, physicochemical, or biological pretreatment and fractionation rather than distillation. Cellulose and hemicellulose can provide fibers, fermentable sugars, furans, organic acids, and related intermediates, whereas lignin can provide phenolic compounds, functionalized oligomers, aromatic intermediates, and carbon-rich materials [2,12,13].

Table 4 summarizes the major characteristics relevant to this comparison.

A particularly important difference is the high oxygen content of biomass. Petroleum processing frequently involves cracking, hydrogenation, and the removal of sulfur, nitrogen, metals, and other undesirable components. Biomass conversion must instead manage oxygen-rich functional groups while preserving valuable molecular structures where possible. Severe deoxygenation can increase hydrogen consumption and remove biomass carbon as CO or CO_2_. Consequently, selective fractionation and controlled conversion are often more important than reproducing petroleum-style cracking.

The two systems also differ in their treatment of heavy or residual fractions. Petroleum vacuum residues and asphaltene-rich streams may be upgraded through thermal or catalytic processes or directed toward lower-value applications. Biomass processing can generate lignin-rich solids, char, aqueous organic compounds, and mineral residues. Although lignin and asphaltenes are both heterogeneous, carbon-rich fractions that present upgrading challenges, they are not chemically equivalent. Lignin is an oxygen-rich aromatic biopolymer containing ether and carbon–carbon linkages, whereas asphaltenes comprise complex petroleum-derived aromatic and aliphatic structures containing variable amounts of sulfur, nitrogen, oxygen, and metals. Their reaction pathways, catalyst requirements, and product distributions therefore differ substantially.

The S-OIL Shaheen Project illustrates industrial integration within a crude-to-chemicals context, particularly the coordination of feedstock conversion, product diversification, and value creation. It is not, however, a biorefinery or evidence of fractal engineering [29,31].

The analogy should therefore be applied at the systems-engineering level—fractionation, conversion, separation, residue upgrading, energy integration, product diversification, and plant-wide optimization—rather than at the level of feedstock chemistry. Similarly, the branching pathways shown in Figure 4 represent hierarchical conversion and product diversification; they do not demonstrate mathematical self-similarity, scale invariance, or a measurable fractal dimension. Any advantage of a fractal-inspired or hierarchical configuration must be established independently through transport and reaction modeling, mass and energy balances, process simulation, experimental comparison, techno-economic analysis, and life-cycle assessment.

### 4.4. Lignin and Asphaltenes: Similarities and Differences

Lignin and petroleum asphaltenes are sometimes compared because both occur as chemically complex fractions that can present significant upgrading challenges. This comparison can be useful at the level of process strategy, but the two materials should not be treated as chemically or structurally equivalent.

Lignin is a heterogeneous, oxygen-rich aromatic biopolymer derived from phenylpropanoid units in plant cell walls. Its structure contains aromatic rings connected through a variety of ether and carbon–carbon linkages and includes substantial hydroxyl and methoxy functionality. Lignin structure also varies with biomass species, isolation method, and processing history [3,4,25].

Asphaltenes, in contrast, are conventionally defined as a petroleum solubility fraction that is insoluble in low-molecular-weight paraffinic solvents such as n-heptane but soluble in aromatic solvents such as toluene. They comprise heterogeneous aromatic-rich molecules containing alkyl groups and varying quantities of sulfur, nitrogen, oxygen, and metals. Their aggregation and phase behavior are important considerations in petroleum production and processing [30,32].

The useful analogy therefore lies not in molecular equivalence but in their process roles: both can represent heterogeneous, carbon-rich fractions whose efficient utilization requires appropriate separation, characterization, conversion, and upgrading strategies. Their chemical structures, oxygen and heteroatom contents, molecular associations, solubility behavior, reaction mechanisms, and catalyst requirements nevertheless differ substantially.

Accordingly, petroleum residue-upgrading strategies may provide useful conceptual lessons for lignin valorization—particularly the principle of converting a difficult or lower-value stream into higher-value products—but petroleum hydrocracking or asphaltene conversion should not be presented as a direct technological model for lignin conversion.

Overall, the principal lesson from petroleum refining is integration rather than imitation. Petroleum refineries demonstrate the value of coordinated fractionation, conversion, residue upgrading, energy integration, product diversification, and plant-wide optimization. Adapting these principles to the fundamentally different chemistry and supply characteristics of lignocellulosic biomass provides a more defensible foundation for the integrated B2C framework developed in this paper.

## 5. Integrated Fractal-AI and Industry 5.0 Framework

The proposed framework integrates three complementary concepts: fractal-inspired design, artificial intelligence (AI), and Industry 5.0. These concepts perform different functions and should not be treated as interchangeable. Fractal-inspired design provides principles for organizing physical structures and process networks; AI provides tools for characterization, prediction, optimization, and control; and Industry 5.0 establishes broader objectives related to sustainability, resilience, and human-centered manufacturing.

Their integration therefore connects architecture, intelligence, and purpose. In a future biorefinery, hierarchical structures may improve material organization and transport; AI may characterize these structures and optimize their operation; and Industry 5.0 objectives determine whether the resulting system provides meaningful improvements in resource efficiency, environmental performance, resilience, and human oversight. Their complementary functions are summarized in Table 5.

This distinction is important because neither hierarchical architecture nor AI implementation alone demonstrates improved sustainability. The proposed framework therefore requires structural descriptors, AI performance metrics, and engineering and sustainability indicators to be evaluated independently and then integrated at the system level.

### 5.1. Fractal-Inspired Material and Reactor Architecture

Fractal-inspired design may be applied at several physical scales within a biorefinery, ranging from catalyst pore structures to reactor channels and fluid-distribution networks. The objective is not to reproduce ideal mathematical fractals but to use selected principles—such as hierarchical organization, branching, multiscale connectivity, and modularity—to address specific transport and reaction requirements.

At the material scale, hierarchical catalysts contain interconnected pores of different sizes. Larger pores can facilitate bulk transport, while smaller pores can provide greater internal surface area and access to catalytic sites. Such architectures may be particularly relevant to biomass-derived molecules and lignin fragments, whose size and chemical heterogeneity can cause diffusion limitations in predominantly microporous catalysts [33,34].

Hierarchical porosity does not necessarily produce superior catalytic performance. Its effectiveness depends on accessible pore volume, pore-size distribution, connectivity, tortuosity, pore constrictions, active-site distribution, molecular dimensions, adsorption behavior, reaction kinetics, and resistance to fouling. Increasing structural complexity may enlarge the nominal surface area while simultaneously creating inaccessible regions, diffusion barriers, weak mechanical structures, or greater manufacturing cost. Candidate catalyst architectures must therefore be evaluated through adsorption measurements, three-dimensional imaging, diffusion analysis, reaction experiments, and stability testing.

At the reactor and equipment scales, hierarchical branching networks can distribute or collect fluids through successive channel levels. Similar arrangements occur in trees, lungs, vascular systems, and river networks, where branching connects transport pathways operating across different scales. Engineered branching manifolds have been investigated for microfluidic devices, heat exchangers, catalytic reactors, fuel cells, and other systems requiring controlled fluid distribution [7,33].

**Figure 5:**
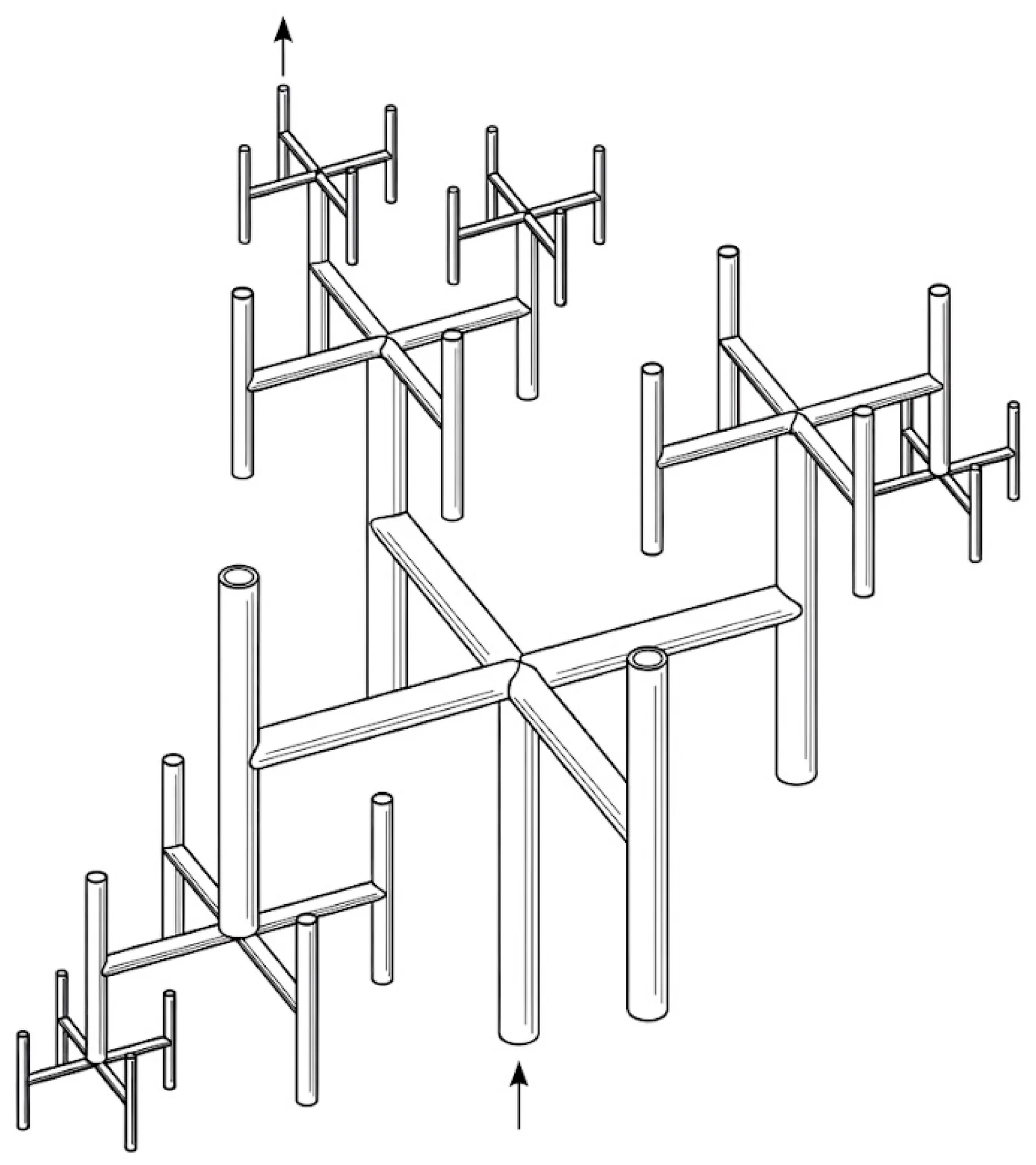
Fractal cascade for fluid distribution and collection, reproduced from [35]. The arrows indicate the direction of fluid flow through the cascade.

Figure 5 illustrates a representative fractal cascade architecture consisting of recursively branching conduits for fluid distribution and collection [35].

The repeated branching pattern shown in Figure 5 represents a fractal-inspired physical architecture in which recursively smaller conduits are distributed across successive channel levels for fluid distribution and/or collection [35]. However, branching alone does not demonstrate mathematical self-similarity, scale invariance, or a measurable fractal dimension. A quantitative description requires explicit specification of channel lengths, diameters, branching ratios, branching angles, and the number and scale range of branching levels.

The engineering performance of a branching network should be evaluated using pressure drop, flow uniformity, residence-time distribution, pumping-energy demand, mixing, heat- and mass-transfer coefficients, susceptibility to blockage, and manufacturing complexity. Computational fluid dynamics can support comparison of alternative geometries, but simulations should be verified through physical flow experiments. Comparisons with conventional manifolds should use the same flow rate, fluid properties, reactor volume, boundary conditions, and performance criteria.

Similar design principles may be applied to heat exchangers and modular processing units. Multiscale channels and extended surfaces may increase heat-transfer area, while repeated functional modules may provide operational flexibility when biomass composition, availability, or product demand changes. These potential advantages must be balanced against pressure loss, fouling, cleanability, maintenance, mechanical reliability, and scale-up requirements.

Additive manufacturing may facilitate the production of complex catalyst supports, flow channels, and reactor components that would be difficult to fabricate using conventional methods. Nevertheless, print resolution, surface roughness, dimensional accuracy, material compatibility, cleanability, mechanical strength, and production cost remain important constraints. The preferred architecture is therefore not necessarily the geometry with the greatest complexity or the largest number of branches, but the design that achieves the best balance among transport performance, reaction efficiency, energy demand, manufacturability, reliability, and cost.

These material and reactor architectures constitute the physical-design layer of the proposed framework. Artificial intelligence can subsequently assist in characterizing their structures, generating candidate geometries, optimizing operating conditions, and controlling their performance. Structural characteristics and AI-enabled operation should nevertheless be evaluated independently so that geometric complexity is not confused with demonstrated engineering improvement.

### 5.2. AI-Enabled Design and Operation

Artificial intelligence provides the analytical and operational capabilities needed to design and manage complex biorefinery systems. Its role is distinct from that of fractal-inspired design: fractal-inspired principles concern physical and organizational architecture, whereas AI supports characterization, prediction, optimization, monitoring, and control. The application of AI does not make a system fractal, nor does it independently demonstrate improved engineering or environmental performance.

At the material scale, computer vision can analyze images obtained by SEM, TEM, AFM, and micro-CT to identify fibers, particles, pores, interfaces, and morphological features. It can also assist image segmentation and the extraction of scale-dependent descriptors. Such measurements should be compared with independent experimental or manually validated results because segmentation choices, image resolution, and preprocessing can strongly influence the calculated structural parameters.

Machine-learning models can relate biomass composition and operating variables to conversion, selectivity, product yield, and quality. These methods may be particularly useful for lignocellulosic biomass because moisture, ash, cellulose, hemicellulose, lignin, and extractive contents vary among feedstocks and batches. Models intended for predictive use should be evaluated with independent test data covering the expected range of feedstock compositions and operating conditions.

AI can also support experimental and structural design. Bayesian optimization can guide the selection of catalyst composition, temperature, pressure, residence time, solvent, and other process variables while reducing the number of experiments needed to identify promising operating regions. Generative-design methods can propose catalyst supports, pore networks, reactor channels, and branching manifolds subject to specified geometric, transport, and manufacturing constraints. Predicted optima and generated structures nevertheless require confirmation through mechanistic modeling, fabrication, and physical testing.

At the process scale, digital twins can combine mechanistic models, sensor data, and operating histories to represent the dynamic behavior of interconnected process units. Predictive control can use this information to coordinate feed rates, reactor conditions, separation operations, heat integration, and product routing. Reinforcement learning may offer an additional approach to sequential process optimization, but it should first be evaluated in constrained simulations or validated digital environments because unconstrained exploration is unsuitable for safety-critical industrial systems.

Anomaly-detection methods can identify operating patterns associated with fouling, catalyst deactivation, equipment degradation, or sensor failure. Their practical value depends on their ability to detect abnormal conditions without producing excessive false alarms or missed detections. Validation should therefore use representative historical failures, controlled experiments, or pilot-scale data.

Table 6 summarizes the principal AI methods considered in this framework, their potential biorefinery applications, and the independent validation required before practical implementation.

AI-generated predictions, geometries, and control policies should not be accepted solely because they improve a computational objective. They must also satisfy physical laws, process constraints, safety requirements, manufacturability, and economic and environmental criteria. Hybrid models that combine mechanistic knowledge with data-driven methods may be especially valuable when available datasets are limited.

Accordingly, AI should be treated as an enabling set of tools rather than as evidence that a system is fractal, sustainable, or technically superior. Its contribution must be demonstrated through improved predictive accuracy, reduced experimental effort, more stable operation, lower energy or resource consumption, improved product quality, or other measurable outcomes. These capabilities should ultimately support the sustainability, resilience, and human-centered objectives of Industry 5.0 discussed in the following section.

### 5.3. Industry 5.0 Objectives

Industry 5.0 provides the broader objectives against which the proposed biorefinery framework should be evaluated. Three objectives are particularly relevant: sustainability, resilience, and human-centered operation [36,37].

Sustainability requires more than maximizing product yield. Relevant indicators include biomass carbon utilization, greenhouse-gas emissions, energy and water consumption, waste generation, chemical and solvent requirements, material circularity, and recovery of valuable process streams. Consequently, claims of improved sustainability should be supported by quantitative metrics and, where appropriate, life-cycle assessment, techno-economic analysis, and mass and energy balances.

Resilience refers to the capacity of the biorefinery to maintain or recover its functions under changing conditions. This is particularly important for biomass processing because feedstocks vary in composition, moisture, ash content, availability, and geographic distribution. Modular processing, flexible product routing, predictive monitoring, and adaptive process control may enable a facility to respond more effectively to feedstock variability, supply disruptions, equipment faults, and changes in product demand.

Human-centered operation recognizes that AI and automation should support rather than eliminate responsible human decision-making. Relevant considerations include operator safety, interpretable decision support, explainability of AI recommendations, workforce training, ergonomic operation, and appropriate human supervision of automated systems. Safety-critical decisions should remain subject to suitable engineering safeguards and human oversight.

The Industry 5.0 component therefore supplies the performance objectives and constraints of the framework: a technically optimized biorefinery is not necessarily an Industry 5.0 system unless its operation also addresses sustainability, resilience, and human-centered considerations.

### 5.4. Enabling Digital and Manufacturing Technologies

Several additional technologies can support implementation of the proposed framework, but they should not themselves be described as fractal technologies.

The Internet of Things (IoT) provides distributed sensing, communication, and data acquisition across equipment and process units. These data can support AI models, digital twins, predictive maintenance, and process control [38].

Digital twins integrate physical-process information, mathematical models, sensor data, and operating history to represent and predict system behavior. Their principal role is therefore dynamic process representation rather than fractal design itself.

Robotics can perform physical operations such as material handling, sampling, inspection, maintenance, and hazardous tasks. Robotics may interact with AI-based perception and control but does not inherently involve fractal principles.

Additive manufacturing can fabricate complex catalyst supports, porous structures, reactor components, and flow networks that may be difficult to manufacture conventionally. It is particularly relevant to fractal-inspired engineering because it can translate computationally designed hierarchical geometries into physical components. Nevertheless, additive manufacturing itself is not inherently fractal.

Blockchain and related distributed-ledger technologies may support feedstock provenance, chain-of-custody information, certification, and product traceability where such functions provide practical value. Their inclusion should be based on a defined traceability requirement rather than an assumed connection to fractal geometry.

Accordingly, IoT, digital twins, robotics, additive manufacturing, and blockchain should be regarded as enabling technologies that may support implementation of the framework. Their presence does not establish either fractality or improved sustainability.

### 5.5. Integrated Framework for Biomass-to-Chemicals

The relationships among these elements can be summarized as a closed-loop engineering framework:

Variable biomass feedstock → characterization → hierarchical fractionation and conversion → products → performance measurement → AI-assisted optimization and control → process adaptation.

Within this framework, fractal-inspired design defines the architecture, AI supplies intelligence, and Industry 5.0 defines the objectives and constraints. Sensor networks and digital twins provide information about the physical system, while advanced manufacturing and robotics provide potential means for implementing optimized structures and operations.

The proposed B2C concept can therefore be viewed as an integrated platform rather than a single conversion technology. Biomass is fractionated into complementary component streams; appropriate catalytic, chemical, biological, or thermochemical processes convert these streams into value-added products; AI assists characterization, prediction, optimization, and control; and system performance is evaluated against quantitative engineering, environmental, resilience, and human-centered criteria.

Importantly, the framework constitutes a design and research hypothesis rather than proof that fractal-inspired architectures inherently improve biorefinery performance. Demonstrating such improvement requires comparison with appropriate conventional designs using measurable outcomes such as product yield, selectivity, carbon efficiency, energy consumption, pressure drop, heat and mass transfer, emissions, process stability, and economic performance.

In this sense, the integration of fractal-inspired design, AI, and Industry 5.0 provides a structured research framework connecting physical architecture, computational intelligence, and industrial purpose.

## 6. Applications to Sustainable Polymer Production

The integrated biorefinery framework described above is particularly relevant to sustainable polymer production because lignocellulosic biomass contains complementary carbohydrate and aromatic fractions that can be converted into polymeric materials, functional additives, and polymer precursors. Rather than treating biomass conversion and polymer production as separate activities, an integrated Biomass-to-Chemicals (B2C) platform can connect feedstock fractionation directly with downstream material applications.

The basic hierarchy can be expressed as:

Biomass fraction → conversion route → intermediate → polymer or material application.

Cellulose, hemicellulose, and lignin require different conversion strategies because of their fundamentally different molecular structures. Consequently, the objective is not to apply a common processing route to all biomass components, but to preserve or transform useful structural features of each fraction and direct the resulting intermediates toward appropriate polymer applications. Representative pathways from lignocellulosic biomass fractions to sustainable polymers and materials are summarized in Table 7.

### 6.1. Cellulose-Based Materials

Cellulose offers two major pathways for sustainable polymer and material production: direct utilization of its polymeric structure and conversion into lower-molecular-weight chemical intermediates.

In the first pathway, cellulose fibers can be mechanically or chemically processed into micro- and nanoscale materials. Cellulose nanofibrils (CNFs) and cellulose nanocrystals (CNCs) combine low density, high specific stiffness, abundant surface hydroxyl functionality, and the ability to form interconnected networks. These characteristics have stimulated their use as reinforcement in polymer composites and as components of films, coatings, membranes, aerogels, and hydrogels [14,15].

The performance of nanocellulose-containing polymers depends not simply on nanoscale dimensions but on dispersion, interfacial adhesion, orientation, aspect ratio, moisture sensitivity, and network formation. Hierarchical organization across the molecular, fibrillar, and macroscopic scales can therefore be an important design consideration. Such structures may be described as hierarchical or fractal-inspired where appropriate, but should not be classified as fractal unless scale-dependent measurements demonstrate fractal behavior.

A second route involves dissolution and regeneration of cellulose. Regenerated cellulose can be processed into fibers, films, membranes, and related functional materials. In this case, the native supramolecular organization of cellulose is disrupted during dissolution and reorganized during regeneration, allowing material structure and properties to be controlled through solvent system, coagulation conditions, orientation, and post-treatment.

Cellulose may also be hydrolyzed to glucose and subsequently converted biologically or catalytically into platform chemicals. This pathway connects the carbohydrate fraction of biomass with a broader family of bio-based polymer precursors.

### 6.2. Hemicellulose-Derived Polymer Pathways

Hemicellulose provides complementary routes to sustainable polymers because it contains heterogeneous C5 and C6 carbohydrate structures that can either be chemically modified as polysaccharides or depolymerized into sugars and platform chemicals.

Pentose-rich hemicellulose streams can be hydrolyzed to sugars such as xylose and subsequently dehydrated to furfural. Furfural and its derivatives provide renewable intermediates for furan-containing chemicals, resins, and polymer precursors. This pathway is particularly relevant to B2C because it transforms a biomass fraction that may otherwise have relatively low value into a source of functional chemical building blocks [16].

An important chemical distinction should be maintained: furfural is primarily associated with pentose-rich hemicellulose streams, whereas 5-hydroxymethylfurfural (HMF) is predominantly obtained from hexose sugars. The two furan platforms therefore originate from related but distinct carbohydrate-conversion pathways.

Hemicelluloses can also be retained as macromolecules and chemically modified through esterification, etherification, oxidation, crosslinking, or incorporation into multicomponent systems. Modified xylans, mannans, and related polysaccharides can be used in films, coatings, barrier materials, and hydrogels. Their practical performance depends on molecular weight, substitution, crosslinking, moisture sensitivity, compatibility with other polymers, and processing conditions [17].

### 6.3. Lignin-Based Polymers and Functional Materials

Lignin is particularly important to sustainable polymer production because its aromatic structure distinguishes it from the carbohydrate fractions of biomass. Rather than simply replacing petroleum-derived polymers on a mass-for-mass basis, lignin valorization seeks to exploit its aromatic functionality in applications where phenolic and carbon-rich structures provide specific value.

One pathway involves the recovery, fractionation, and modification of lignin for phenolic resins and adhesives. Because lignin contains phenolic structures, appropriately processed lignin can partially replace petroleum-derived phenolic components in resin formulations. Performance depends strongly on lignin source, molecular-weight distribution, functional-group content, condensation degree, and chemical modification [18].

A second pathway uses functionalized lignin or lignin-derived oligomers in epoxy and polyurethane systems. Hydroxyl functionality can be modified or exploited to produce reactive components for thermosetting polymers. Chemical functionalization may improve reactivity, compatibility, processability, and incorporation into polymer networks. The extent of petroleum-derived component substitution must nevertheless be evaluated together with mechanical, thermal, rheological, and durability properties [19,20].

A third route exploits lignin’s high carbon content and aromatic character to produce carbon-rich functional materials. Appropriate stabilization, carbonization, and structural control can produce carbon fibers, porous carbons, and conductive carbonaceous materials. Such products may serve as reinforcement, adsorbents, electrodes, or functional fillers in polymer composites [21].

Lignin valorization therefore illustrates an important principle of integrated biorefining: a fraction traditionally used primarily for process energy can potentially be directed toward higher-value materials when its composition, purity, molecular structure, and processing characteristics satisfy the requirements of the intended application.

### 6.4. Biomass-Derived Platform Chemicals for Polymer Synthesis

A complementary strategy is to depolymerize biomass carbohydrates and subsequently convert the resulting sugars into platform chemicals suitable for conventional or emerging polymerization routes.

Cellulose- and hemicellulose-derived carbohydrates can provide intermediates such as organic acids, diols, furans, and related oxygenated compounds. Depending on the conversion pathway, these intermediates can serve as monomers or monomer precursors for polyesters, polyamides, polyurethanes, furan-based polymers, and other bio-based polymer systems [16,39].

This pathway differs conceptually from direct use of cellulose, hemicellulose, or lignin as polymeric materials. It involves progressively greater molecular transformation:

Biomass → fractionation → carbohydrates → sugars → platform chemicals → monomers → polymers.

The degree of processing should therefore be justified by the value and performance of the resulting products, because extensive depolymerization, purification, and chemical upgrading may increase energy demand, solvent use, and carbon losses.

Mixed or incompletely separated biomass streams may also be appropriate for selected conversion processes. However, feedstock composition, impurities, ash, inhibitors, and competing reactions become particularly important when high-purity monomers are required. The optimum degree of fractionation consequently depends on the intended polymer pathway.

### 6.5. Hierarchical Integration from Biomass to Polymer

The significance of the proposed B2C framework lies in connecting biomass fractionation with polymer production through a series of chemically appropriate hierarchical pathways rather than forcing all biomass components through a single conversion route.

At the first level, lignocellulosic biomass is separated into cellulose-, hemicellulose-, lignin-, and extractive-rich streams. At the second level, each fraction is processed according to its molecular characteristics. At the third level, these processes generate intermediates such as nanocellulose, regenerated cellulose, sugars, furans, phenolic fractions, functionalized lignin, and platform chemicals. At the final level, the intermediates are incorporated into or converted into polymers and functional materials.

This hierarchy can be summarized as:

Lignocellulosic biomass → Cellulose → nanocellulose/regenerated cellulose/sugars → composites, films, fibers, membranes, hydrogels, and polymer precursors → Hemicellulose → modified polysaccharides/sugars/furans → films, barriers, hydrogels, resins, and furan-based polymer precursors → Lignin → phenolic fractions/functionalized oligomers/carbon materials → adhesives, thermosets, composites, and functional carbon materials.

The hierarchy is fractal-inspired at the system-design level because a common biomass resource branches into multiple fractions, intermediates, and product pathways. This organizational analogy should not, however, be interpreted as evidence that the chemical conversion network itself exhibits mathematical fractality.

The principal advantage of this integrated perspective is therefore not fractality itself, but the possibility of matching the intrinsic chemistry of each biomass fraction with suitable high-value polymer applications. The success of these pathways should ultimately be evaluated using measurable criteria—including conversion yield, carbon efficiency, polymer properties, process energy demand, recyclability, environmental impacts, and economic performance.

### 6.6. Implications for Sustainable Polymer Manufacturing

For sustainable polymer production, renewable feedstock origin alone is insufficient to establish environmental superiority. A bio-based polymer pathway may require substantial energy, chemicals, solvents, purification, or downstream processing. Conversely, retaining useful macromolecular structures—as in nanocellulose, regenerated cellulose, or functionalized lignin—may sometimes reduce the extent of molecular reconstruction required to produce useful materials.

The proposed framework therefore favors value-preserving conversion where technically appropriate: cellulose can retain its fibrillar or polymeric structure, hemicellulose can be utilized either as a functional polysaccharide or converted selectively into chemical intermediates, and lignin can retain useful aromatic functionality rather than necessarily being completely depolymerized.

This approach connects the B2C concept directly with sustainable polymer manufacturing. Biomass fractionation determines the available molecular resources; conversion determines the intermediates; and polymer engineering determines how those intermediates can provide useful material properties. The resulting hierarchy—biomass fraction → conversion route → intermediate → polymer application—provides a focused framework for evaluating the role of lignocellulosic biorefineries in future sustainable polymer production.

## 7. Quantitative Evaluation and Validation

### 7.1. Representative Mass Balance

To illustrate the quantitative evaluation of biomass fractionation without combining data from unrelated studies, a representative mass balance was constructed from the pilot-scale sugarcane-bagasse study of Rocha et al. [40]. The process consisted of steam-explosion pretreatment followed by alkaline delignification and acid precipitation of solubilized lignin. The untreated bagasse contained approximately 45.5% cellulose, 27.0% hemicellulose, 21.1% lignin, 4.6% extractives, and 2.2% mineral matter on a dry basis.

On the basis of 100 kg of dry sugarcane bagasse, these values correspond approximately to 45.5 kg cellulose, 27.0 kg hemicellulose, 21.1 kg lignin, 4.6 kg extractives, and 2.2 kg mineral matter. The slight excess over 100 kg results from analytical/component reporting and rounding and should not be artificially normalized, because doing so would alter the published composition.

During steam explosion, 82.6% of the pentosan fraction was solubilized, while 90.2% of cellulose and 87.0% of lignin were recovered in the solid fraction. Subsequent alkaline delignification solubilized approximately 91% of the lignin and 72.5% of the pentosans remaining in the steam-exploded solids, producing a cellulose-rich pulp containing almost 90% cellulose. Acidification of the alkaline liquor recovered lignin corresponding to 48.3% of the lignin initially present in the raw material. Across the overall process, approximately 22% of cellulose, 26% of pentosans, and 14% of lignin were reported as losses [40].

The approximate values in Table 8 are obtained directly from the published percentages. For example, cellulose recovered 45.5 kg × 0.78 ≈ 35.5 kg; pentosans accounted for 27.0 kg × 0.74 ≈ 20.0 kg.

Similarly, the reported overall lignin loss gives 21.1 kg × (1 − 0.14) ≈ 18.1 kg accounted for.

Thus, approximately 18.1 kg of the original 21.1 kg lignin is accounted for after subtracting the reported overall loss, of which approximately 10.2 kg was specifically recovered by acid precipitation. These calculations should be regarded as a component balance, rather than a complete plant-wide mass balance.

This example is particularly appropriate for the present framework because it demonstrates why quantitative validation must distinguish between fractionation efficiency and total resource utilization. Steam explosion effectively separated much of the pentosan fraction, and alkaline treatment produced a cellulose-rich pulp while enabling recovery of a substantial lignin fraction. Nevertheless, measurable losses remained: approximately 22% of cellulose, 26% of pentosans, and 14% of lignin. Rocha et al. specifically identified process modifications, including improved condensation and fewer washing steps, as opportunities to increase material recovery [40].

These losses are important in the context of the proposed B2C framework. A process should not be described as resource-efficient merely because it produces a high-purity cellulose fraction or recovers lignin. All major biomass-carbon streams must be considered. Quantitative optimization should therefore seek simultaneously to increase recovery of cellulose, hemicellulose-derived products, and lignin-derived products while reducing uncharacterized aqueous organics, gaseous carbon losses, and solid residues.

Importantly, the Rocha et al. dataset should not be extended artificially to calculate energy efficiency, carbon efficiency, or polymer yield because the required complete stream compositions, energy inputs, and downstream conversion data were not reported. Those quantities should be obtained from a separate, explicitly identified process model or experimental dataset rather than estimated by combining unrelated literature values. This approach is consistent with recommended practice for lignin-first and integrated biorefinery mass balances, where system boundaries and definitions of fraction and product yields must be explicitly specified.

To extend such fractionation data to the overall metrics defined in Section 7.2, downstream datasets should provide, as applicable, intermediate-stream composition and recovery, catalytic or biochemical conversion, product selectivity and yield, separation and purification recovery, and polymerization conversion and isolated polymer yield. The associated consumption of hydrogen, catalysts, solvents, water, electricity, and thermal energy should also be reported when carbon and energy efficiencies are evaluated. Ideally, these downstream data should be obtained from an integrated experimental study or process model using compatible feed compositions, operating conditions, mass bases, and system boundaries. Combining unrelated literature datasets without such compatibility could produce an apparently complete mass balance that does not represent a physically consistent process.

### 7.2. Performance Equations

Quantitative comparison requires clearly defined performance indicators and system boundaries. At minimum, mass closure, carbon efficiency, product yield, and energy efficiency should be reported.

Mass closure evaluates whether the measured or modeled input mass is accounted for in the output streams, as defined in Equation (3):

(3) $$M_{\mathrm{closure}}=\frac{\sum m_{\mathrm{out}}}{\sum m_{\mathrm{in}}}\times 100\%$$

In Equation (3), *M*_closure_ denotes mass closure (in percent), and *m*_out_ and *m*_in_ are the masses of the specified output and input streams, respectively. All streams included in the calculation should be identified. Water and recycled streams may dominate total mass flows and can obscure biomass conversion; therefore, both an overall process balance and a dry-biomass or organic-matter balance may be useful.

Carbon efficiency measures the fraction of biomass-derived carbon recovered in the target product portfolio (Equation (4)):

(4) $$\eta_{C}=\frac{m_{C,\mathrm{products}}}{m_{C,\mathrm{biomass}}}\times 100\%$$

In Equation (4), *η*_C_ denotes carbon efficiency (in percent); *m*_C,products_ and *m*_C,biomass_ are the masses of biomass-derived carbon in target products and in the biomass input, respectively. For multiple valuable products, the numerator is the sum of biomass-derived carbon contained in all designated valuable product streams.

Carbon incorporated from external sources should be accounted for separately. For example, externally supplied carbon-containing solvents or reagents should not be counted as biomass-derived carbon.

Product yield on a dry-biomass basis is defined by Equation (5):

(5) $$Y_{\mathrm{product}}=\frac{m_{\mathrm{target}\;\mathrm{product}}}{m_{\mathrm{dry}\;\mathrm{biomass}}}\times 100\%$$

For component-specific conversion, the denominator in Equation (5) should be the mass of the corresponding biomass component; a lignin-derived product yield, for example, uses the mass of lignin entering conversion.

In Equation (5), *Y*_product_ is product yield (in percent), *m*_target product_ is target-product mass, and *m*_dry biomass_ is dry feedstock mass. The basis must therefore accompany every reported yield.

A simplified energy efficiency is given by Equation (6):

(6) $$\eta_{E}=\frac{\sum E_{\mathrm{useful}\;\mathrm{products}}}{E_{\mathrm{biomass}}+\sum E_{\mathrm{external}}}\times 100\%$$

In Equation (6), *η*_E_ is energy efficiency (in percent), *E*_useful products_ denotes useful product energy, *E*_biomass_ is the energy content of the biomass, and *E*_external_ includes imported energy crossing the selected system boundary. For chemical or polymer production, energy efficiency should be considered together with carbon efficiency, product yield, and economic and environmental indicators.

The treatment of hydrogen, electricity, steam, solvents, catalysts, water, and recycled streams must be explicitly defined. Recycled internal streams should not be repeatedly counted as new inputs, whereas makeup solvent, imported hydrogen, and externally generated electricity or steam should be included when they cross the system boundary.

### 7.3. Comparative Validation of Fractal-Inspired Designs

Validation of fractal-inspired engineering requires more than measuring fractal dimension. The structural modification must be connected experimentally or computationally with the engineering property it is intended to improve. A multiscale validation framework for fractal-inspired biorefinery design is summarized in Table 9.

At the material scale, microscopy, tomography, adsorption measurements, and diffusion or permeation experiments can determine whether a proposed hierarchical pore structure actually increases accessible surface area or improves transport. Fractal dimension may complement these measurements but cannot replace them.

At the catalyst scale, conventional reactor measurements should establish conversion, selectivity, productivity, and stability. A hierarchical catalyst should therefore be compared with an appropriate conventional catalyst having, as far as practical, comparable chemical composition and active-site characteristics. Otherwise, improvements caused by catalyst chemistry could incorrectly be attributed to pore architecture.

At the reactor scale, CFD can evaluate velocity fields, pressure gradients, mixing, heat transfer, and residence-time distributions in conventional and fractal-inspired geometries. Computational predictions should subsequently be tested against physical flow experiments where feasible.

At the process scale, mass and energy balances and process simulation should determine whether improvements observed in an individual catalyst or reactor remain beneficial after separation, recycle, compression, heating, cooling, solvent recovery, and other process requirements are included. Local performance improvement does not necessarily produce plant-wide improvement.

At the economic scale, TEA should evaluate whether additional fabrication complexity, catalyst preparation, instrumentation, computation, or process-control requirements are justified by improvements in yield, productivity, energy use, or product value. Relevant indicators may include capital expenditure, operating expenditure, minimum selling price, net present value, or other appropriate economic measures.

At the environmental scale, LCA should assess whether the proposed system reduces environmental burdens rather than merely transferring them between process stages. Greenhouse-gas emissions, energy and water use, land and resource requirements, chemical consumption, waste generation, and other relevant impact categories should be evaluated using explicitly defined system boundaries and assumptions.

### 7.4. Controlled Comparison with Conventional Systems

A central requirement for validation is the use of an appropriate reference system. Fractal-inspired and conventional configurations should be compared under the same feedstock, kinetic, transport, and operating assumptions. Without such controls, improved performance cannot be attributed to architecture.

For example, comparison of a hierarchical catalyst with a conventional catalyst should control, where possible, catalyst composition, active-phase loading, reaction temperature, pressure, feed composition, space velocity, and reactor configuration. Similarly, comparison of branching and conventional fluid manifolds should use the same total flow rate, fluid properties, inlet and outlet conditions, and relevant geometric constraints.

Relative performance change compared with a conventional reference is calculated using Equation (7):

(7) $$\Delta P=\frac{P_{\mathrm{FI}}-P_{\mathrm{reference}}}{P_{\mathrm{reference}}}\times 100\%$$

In Equation (7), *P*_FI_ and *P*_reference_ are the selected performance metrics for the fractal-inspired and conventional configurations, respectively. For metrics where lower values are preferable, such as pressure drop or energy consumption, the sign convention and interpretation should be stated explicitly.

Multiple performance indicators should generally be considered simultaneously. A branching manifold, for example, may improve flow uniformity while increasing fabrication complexity; a hierarchical catalyst may improve conversion while increasing pressure drop or reducing mechanical stability. Consequently, claims of superiority should be based on the relevant combination of technical, economic, and environmental metrics rather than on a single structural parameter.

### 7.5. Validation Roadmap for the Proposed B2C Framework

Because the present framework is conceptual, its validation should proceed progressively from material characterization to integrated process assessment. First, candidate hierarchical structures should be quantitatively characterized over defined length scales. Second, their transport, reaction, mechanical, or thermal performance should be compared with suitable conventional references. Third, validated component-level results should be incorporated into reactor and process models. Finally, the integrated process should be evaluated using mass and energy balances, TEA, and LCA.

This sequence can be summarized as:

Structure → characterization → engineering performance → controlled comparison → process integration → TEA/LCA → system-level validation.

AI can support each stage through image analysis, surrogate modeling, optimization, digital twins, and process control, but AI predictions should themselves be validated against independent experimental or plant data.

The quantitative framework proposed here therefore establishes how the fractal-inspired B2C concept should be tested rather than claiming performance improvements that have not yet been experimentally demonstrated. This distinction is essential: the scientific value of the proposed framework lies not in assuming that hierarchical or fractal-inspired structures are inherently superior, but in generating measurable hypotheses that can be tested under controlled and reproducible conditions.

## 8. Challenges, Research Priorities, and Future Perspectives

The integration of fractal-inspired design, biomass fractionation, artificial intelligence, and Industry 5.0 provides a potentially useful framework for developing advanced biorefineries. However, the framework remains largely conceptual, and several scientific and engineering challenges must be addressed before its advantages can be established quantitatively. In particular, future research should distinguish carefully between fractal characterization, hierarchical engineering design, AI-assisted optimization, and demonstrated process performance.

The central research question is therefore not whether fractal principles can be identified qualitatively in biomass or industrial systems, but whether specific fractal-inspired architectures can produce measurable improvements relative to appropriate conventional designs. Such improvements must ultimately be demonstrated through controlled experiments, process simulation, scale-up studies, techno-economic analysis, and environmental assessment.

### 8.1. Establishing Quantitative Structure–Performance Relationships

A major challenge is establishing reproducible relationships between structural descriptors and engineering performance. Fractal dimension can quantify certain forms of scale-dependent complexity, but it does not directly determine accessible surface area, diffusivity, permeability, catalytic activity, heat transfer, pressure drop, or mechanical properties. Two structures with similar fractal dimensions may have substantially different pore connectivity, tortuosity, pore-size distributions, surface chemistry, and transport behavior.

Future studies should therefore combine fractal descriptors with conventional structural and engineering measurements. For porous catalysts, for example, fractal dimension may be reported together with pore-size distribution, accessible surface area, connectivity, tortuosity, effective diffusivity, conversion, selectivity, and catalyst stability. For reactor manifolds, branching characteristics should be related quantitatively to flow uniformity, pressure drop, residence-time distribution, heat transfer, and pumping requirements.

An important research objective is consequently to determine when a fractal descriptor provides information beyond established morphological descriptors. If fractal dimension does not improve interpretation, prediction, or design, its inclusion adds little engineering value.

### 8.2. Standardization of Fractal Characterization

Fractal dimensions obtained using different experimental or computational techniques do not necessarily describe the same physical property. Box-counting analysis of a two-dimensional microscopy image, surface-fractal analysis from adsorption measurements, and mass-fractal analysis from small-angle scattering probe different structural characteristics and length scales. Numerical values obtained by these methods should therefore not be compared directly without establishing their physical meaning.

Future studies should report, at minimum, the characterization method, spatial resolution, scale interval, preprocessing and segmentation procedures, fitting method, goodness of fit, uncertainty, and physical interpretation of the reported dimension. Evidence of a linear relationship on a log–log plot alone is insufficient if the scaling interval is too narrow or strongly dependent on image-processing parameters.

For engineering applications, a standardized reporting framework would improve reproducibility and facilitate comparison among biomass structures, catalysts, membranes, porous polymers, and reactor components. Particular attention should be given to identifying the finite range of scales over which statistical self-similarity is actually observed.

### 8.3. Scale-Up of Hierarchical and Fractal-Inspired Structures

Many hierarchical structures can be demonstrated at laboratory scale but become difficult or expensive to manufacture at industrial scale. Complex catalyst pore architectures, branching fluid networks, and multiscale heat-transfer surfaces may require specialized fabrication techniques and may introduce additional mechanical, hydraulic, or maintenance constraints.

For example, a complex branching manifold may improve flow distribution while increasing fabrication cost or susceptibility to fouling. A hierarchical catalyst may improve molecular transport while reducing mechanical strength or increasing synthesis complexity. Similarly, a heat exchanger with highly structured channels may enhance heat transfer but become more difficult to clean.

Hydraulic resistance represents an additional constraint in the scale-up of multilevel branching networks. Although successive branching may improve flow distribution, reductions in channel dimensions can substantially increase pressure drop and pumping-energy requirements, particularly for viscous or solids-containing biomass streams. The magnitude of this penalty depends on channel geometry, branching ratio, fluid properties, flow regime, and the number of branching levels. Consequently, increasing the number of hierarchical levels does not necessarily improve overall process performance. Flow uniformity and transport enhancement must instead be balanced against pressure drop, pumping power, fouling susceptibility, and practical operating requirements.

Future research should therefore consider manufacturability, mechanical integrity, fouling, cleanability, maintenance, catalyst regeneration, and lifetime performance from the beginning of the design process. Additive manufacturing may expand the range of realizable geometries, but the performance advantages of complex structures must justify their additional fabrication and operating requirements.

### 8.4. Feedstock Variability and Process Flexibility

Lignocellulosic biomass presents a challenge that is less pronounced in many conventional chemical processes: substantial variation in chemical composition and physical properties. Cellulose, hemicellulose, lignin, extractives, ash, moisture, particle morphology, and contaminant levels may vary with biomass species, plant tissue, growing conditions, harvesting, storage, and preprocessing.

Consequently, a biorefinery optimized for a single well-characterized feedstock may not perform optimally when feedstock properties change. Future B2C systems should therefore incorporate feedstock characterization, flexible fractionation, adaptive operating conditions, and alternative product-routing strategies.

This is an area in which AI may provide particular value. Models incorporating feedstock composition and process data could potentially predict fractionation behavior, conversion yield, product quality, and operating requirements. However, such models should be trained and tested across sufficiently diverse feedstocks and operating conditions. A model validated only on data closely resembling its training set cannot be assumed to provide reliable predictions for new biomass resources.

### 8.5. Data Quality, AI Validation, and Digital Twins

The effectiveness of AI-assisted biorefineries will depend strongly on the quality, quantity, and representativeness of available data. Laboratory datasets are frequently small, generated under restricted operating conditions, and collected using different analytical procedures. These limitations can produce models that perform well statistically but fail when applied to different feedstocks, equipment, or operating regimes [9].

Future studies should therefore emphasize data provenance, uncertainty, external validation, and physically meaningful variables. Hybrid approaches combining mechanistic process knowledge with data-driven models may be particularly valuable when experimental data are limited.

For variable feedstocks such as kenaf and sugarcane bagasse, hybrid mechanistic–data-driven models could explicitly incorporate batch-specific properties such as moisture and ash content as model inputs or state variables. The mechanistic component can impose physical constraints, including mass and energy balances, while the data-driven component can learn nonlinear relationships among feedstock properties, operating conditions, and process responses. For example, changes in moisture content may alter drying and thermal-energy requirements, whereas variations in ash content may influence fouling, catalyst performance, and downstream separation. Incorporating these measured properties into hybrid models could support batch-specific adjustment of operating conditions while reducing reliance on purely empirical correlations derived from narrowly controlled laboratory datasets.

Digital twins present similar challenges. A digital twin should not simply reproduce historical process data but should provide a validated representation capable of responding realistically to changing operating conditions. Validation against independent pilot- or plant-scale measurements is therefore essential [41,42].

For safety-critical process control, explainability and uncertainty become especially important. AI recommendations should operate within defined engineering and safety constraints, with appropriate human supervision, rather than functioning as unconstrained optimization systems.

### 8.6. Carbon, Energy, and Economic Performance

Improvement at one scale does not necessarily translate into improvement at the integrated-process level. A catalyst that increases product yield may require greater hydrogen consumption; a new fractionation process may improve lignin recovery while increasing solvent-recovery energy; and a complex reactor architecture may improve transport while requiring more expensive fabrication.

Future evaluations should therefore extend beyond isolated performance indicators. At minimum, promising configurations should be assessed using mass closure, carbon efficiency, product yield, energy requirements, and process losses, as discussed in Section 7. At more advanced stages, these assessments should be integrated with TEA and LCA.

Particular attention should be given to the definition of system boundaries. Hydrogen production, electricity, steam, solvent manufacture and recovery, water consumption, catalyst preparation, waste treatment, and transportation can materially affect overall performance. Comparisons should use equivalent boundaries and assumptions.

For polymer applications, evaluation should additionally consider whether a biomass-derived material provides the required functionality and lifetime. Renewable carbon content alone does not establish sustainability if the resulting polymer requires substantially greater processing energy, has poor durability, or creates difficult end-of-life problems.

### 8.7. Circularity and End-of-Life Considerations

Sustainable polymer production requires attention not only to renewable feedstocks but also to the fate of materials after use. Bio-based does not necessarily mean biodegradable, recyclable, or environmentally preferable under all conditions. Polymer chemistry, additives, composite structures, contamination, collection systems, and recycling technologies determine actual end-of-life options [39].

Future B2C research should therefore incorporate end-of-life considerations during material design. Depending on the application, desirable strategies may include mechanical recycling, chemical recycling, biodegradation under defined conditions, recovery of functional components, or controlled energy recovery when higher-value options are impractical.

This consideration is particularly important for multicomponent materials. Nanocellulose reinforcement, lignin-derived additives, coatings, crosslinking agents, and other bio-based components may improve material performance while simultaneously affecting recyclability. Circularity should consequently be evaluated as a system property rather than inferred from renewable feedstock origin.

### 8.8. From Conceptual Framework to Experimental Demonstration

A practical research pathway for the proposed framework should proceed incrementally rather than attempting immediate validation at the complete biorefinery scale. The first stage should identify a specific engineering problem for which hierarchical or fractal-inspired architecture provides a testable hypothesis. The second should quantitatively characterize the proposed structure and compare its performance with a conventional reference. The third should determine whether the observed component-level improvement remains significant when incorporated into reactor and process models.

The resulting progression can be expressed as:

Design hypothesis → structural characterization → controlled experiment → engineering validation → process integration → pilot demonstration → TEA and LCA → industrial assessment.

For example, a hierarchical catalyst could first be compared with a chemically similar conventional catalyst under identical reaction conditions. If improved transport, conversion, or selectivity is demonstrated, the validated kinetic and transport data could then be incorporated into reactor and process models. Only after the system-level effects on energy use, carbon efficiency, product yield, and cost have been established would pilot-scale evaluation be justified.

This approach also provides a rational role for AI. AI can assist structural characterization, experimental design, surrogate modeling, optimization, and process control at each stage, but it should not substitute for experimental evidence.

### 8.9. Future Perspectives

The longer-term opportunity is the development of biorefineries in which feedstock characterization, hierarchical fractionation, component-specific conversion, advanced materials design, and adaptive process operation are integrated within a common quantitative framework. Such systems could respond dynamically to changes in biomass composition and direct cellulose, hemicellulose, lignin, and other fractions toward product pathways that maximize resource utilization and value.

Within this vision, fractal-inspired design provides one possible strategy for organizing structures and transport networks across multiple scales; AI provides tools for analyzing and optimizing increasingly complex systems; and Industry 5.0 provides broader sustainability, resilience, and human-centered objectives. None of these elements alone guarantees superior performance.

The scientific opportunity therefore lies in moving from analogy to measurement, from structural complexity to demonstrated function, and from conceptual integration to quantitative validation. If these requirements are met, fractal-inspired engineering may become a useful component of future B2C biorefineries and sustainable polymer manufacturing. Its ultimate value, however, should be determined by measurable improvements in material performance, resource utilization, carbon and energy efficiency, economic viability, environmental impacts, resilience, and safe human-centered operation.

## 9. Conclusions

This review critically examined fractal-inspired organization as a design perspective for integrated biorefineries producing renewable chemicals, materials, and polymer precursors. Deterministic mathematical fractals provide useful models for understanding recursion, branching, self-similarity, and multiscale organization. Real biomass structures and engineered systems, however, are more appropriately described as statistically fractal over defined scale ranges, hierarchical, or fractal-inspired, depending on the available quantitative evidence. Fractal dimension should therefore be regarded as a structural descriptor rather than a direct measure of transport efficiency or engineering performance.

The proposed framework assigns distinct but complementary roles to three principal elements. Fractal-inspired design provides principles for organizing material structures, catalyst pores, transport networks, process modules, and product pathways across multiple scales. Artificial intelligence supports structural characterization, prediction, optimization, process control, and condition monitoring. Industry 5.0 provides the broader objectives of sustainability, resilience, and human-centered operation. Their integration thus connects architecture, intelligence, and purpose within a common biorefinery framework [36,37].

Petroleum refining provides useful but limited lessons in integrated fractionation, conversion, residue upgrading, heat and energy integration, product diversification, and plant-wide optimization. These principles may inform biorefinery development, but petroleum and lignocellulosic biomass differ fundamentally in composition, molecular structure, conversion chemistry, feedstock supply, and processing requirements. The appropriate lesson is therefore integration rather than imitation. Likewise, Biomass-to-Chemicals (B2C) should be understood here as a unifying framework for connecting biomass fractionation and component-specific valorization with chemical and material production, rather than as a claim that biomass-to-chemicals is itself a concept introduced by this study.

For sustainable polymer production, this integrated perspective provides a hierarchy linking biomass fraction → conversion route → intermediate → polymer or material application. Cellulose can provide nanocellulose, regenerated cellulose, and carbohydrate-derived intermediates; hemicellulose can provide functional polysaccharides and furan-based intermediates; and lignin can provide phenolic compounds, functionalized oligomers, and carbon-rich materials. The objective is to match the intrinsic chemistry and structure of each biomass fraction with suitable high-value applications while improving overall carbon and resource utilization.

The proposed fractal-inspired B2C framework nevertheless remains a testable design hypothesis rather than a demonstrated performance advantage. Its value must be established through controlled comparisons with conventional systems using structural characterization, transport modeling, reaction engineering, mass and energy balances, process simulation, experimental validation, techno-economic analysis, and life-cycle assessment. Comparisons should employ equivalent feedstocks, system boundaries, kinetics, transport conditions, and operating assumptions so that observed improvements can be attributed to architecture rather than to unrelated process differences.

Future research should therefore move from analogy to measurement, from structural complexity to demonstrated function, and from conceptual integration to quantitative validation. Such studies will determine whether fractal-inspired architectures, when combined with validated AI tools and Industry 5.0 objectives, can provide measurable improvements in resource utilization, process efficiency, resilience, environmental performance, and sustainable polymer production.

## Figures and Tables

**Table 1:** Representative deterministic fractals and their engineering analogies.

Mathematical Structure	Principle Illustrated	Possible Design Analogy	Limitation
Cantor construction	Recursive removal	Discontinuous pore distribution	Does not represent a real catalyst
Koch geometry	Boundary enlargement	High-interface structures	Increased boundary does not prove accessibility
Sierpinski carpet	Hierarchical voids	Multilevel planar porosity	Ideal deterministic geometry
Menger sponge	Three-dimensional void hierarchy	Multiscale porous media	Does not predict transport by itself

**Table 2:** Structural descriptors and engineering performance metrics for representative fractal or hierarchical systems.

System	Structural Descriptor	Engineering Performance Metric
Porous catalyst	Pore/surface fractal dimension, pore-size distribution, connectivity	Effective diffusivity, conversion, selectivity
Membrane	Pore structure, connectivity, tortuosity	Permeability, selectivity/rejection, pressure drop
Fluid manifold	Branching architecture, channel-size distribution, connectivity	Pressure drop, flow uniformity, pumping demand
Heat exchanger	Multiscale channel geometry, surface structure	Heat-transfer coefficient, pressure drop, pumping demand
Porous polymer	Pore/network structure, connectivity, surface dimension	Mechanical, thermal, and mass-transport properties

**Table 3:** Representative lignocellulosic resources and potential valorization pathways.

Resource	Distinctive Characteristics	Principal Components	Representative Products
Kenaf	Fast-growing non-wood crop; distinct bast and core fibers	Cellulose, hemicellulose, lignin	Pulp, fibers, nanocellulose, sugars, lignin-derived products, biocomposites
Sugarcane bagasse	Abundant residue from sugar production	Cellulose, hemicellulose, lignin	Pulp, nanocellulose, fermentable sugars, furfural, xylitol, lignin-derived products
Oil-palm residues	Diverse residues including EFB, OPF, OPT, mesocarp fiber, and shells	Cellulose, hemicellulose, lignin, extractives	Fibers, composites, sugars, fuels, chemicals, carbon materials
Black liquor *	Kraft-pulping process stream	Dissolved lignin, carbohydrate-derived organics, extractives, inorganic pulping chemicals	Recovered lignin, energy, chemicals, aromatic and polymer precursors

* Black liquor is not a primary lignocellulosic feedstock but a lignin-rich process stream generated during kraft pulping.

**Table 4:** Major characteristics of petroleum refining and lignocellulosic biorefineries.

Characteristic	Petroleum	Lignocellulosic Biomass
Principal composition	Hydrocarbon-rich complex mixture	Oxygen-rich carbohydrate–lignin matrix
Major structural components	Hydrocarbon classes and boiling-range fractions	Cellulose, hemicellulose, lignin, extractives, and minerals
Feedstock variability	Dependent on crude source and grade	Dependent on species, tissue, season, moisture, ash, storage, and processing history
Primary separation	Primarily physical distillation according to boiling range	Physical, chemical, physicochemical, or biological fractionation
Main conversion challenge	Cracking, molecular rearrangement, heteroatom removal, and upgrading	Cell-wall deconstruction, selective fractionation, depolymerization, and oxygen management
Supply structure	Concentrated and globally traded	Frequently dispersed, seasonal, and regionally collected
Water content	Generally low	Often substantial and variable
Difficult-to-upgrade streams	Heavy oils, vacuum residue, asphaltenes, and coke	Lignin-rich streams, char, aqueous organics, and processing residues
Integration objective	Maximize value across fuels and chemicals	Maximize value across fuels, chemicals, fibers, materials, and energy

**Table 5:** Complementary functions of fractal-inspired design, AI, and Industry 5.0.

Element	Primary Function	Question Answered
Fractal-inspired design	Physical and organizational architecture	How is the system structured?
Artificial intelligence	Characterization, prediction, optimization, and control	How is the system analyzed and operated?
Industry 5.0	Sustainability, resilience, and human-centered objectives	What should the system achieve?

**Table 6:** Representative AI applications and validation requirements in fractal-inspired biorefineries.

AI Method	Representative Application	Required Validation
Computer vision	Biomass and pore-image characterization	Comparison with independent measurements and sensitivity to image processing
Machine learning	Prediction of conversion, yield, selectivity, and product quality	Independent test data, uncertainty analysis, and evaluation across variable feedstocks
Bayesian optimization	Selection of catalysts and operating conditions	Experimental confirmation of predicted optima
Generative design	Design of catalyst supports, flow channels, and reactor manifolds	Transport and reaction modeling, manufacturability assessment, and physical testing
Digital twins	Dynamic representation of process units and integrated operations	Comparison with independent pilot- or plant-scale data
Predictive control	Coordinated control of reactors, separations, energy use, and product routing	Stability, robustness, constraint-handling, and safety testing
Reinforcement learning	Sequential process optimization and adaptive operation	Constrained simulation and supervised testing before physical deployment
Anomaly detection	Detection of fouling, catalyst deactivation, equipment faults, and sensor failure	Validation using historical failures or controlled experimental data

**Table 7:** Representative pathways from lignocellulosic biomass fractions to sustainable polymers and materials.

Biomass Fraction	Representative Intermediate	Polymer or Material Application
Cellulose	Nanocellulose	Reinforcement, films, coatings, hydrogels
Cellulose	Regenerated cellulose	Fibers, films, membranes
Hemicellulose	Furfural and derivatives	Furan-based polymer precursors
Hemicellulose	Modified polysaccharides	Films, barrier materials, hydrogels
Lignin	Phenolic fractions	Phenolic resins and adhesives
Lignin	Functionalized oligomers	Epoxy and polyurethane components
Lignin	Carbon-rich materials	Carbon fibers, carbon materials, conductive fillers
Mixed biomass streams	Platform chemicals	Bio-based polyesters and related polymers

**Table 8:** Representative component balance for pilot-scale fractionation of 100 kg dry sugarcane bagasse.

Component or Stream	Initial Amount (kg)	Reported Result	Calculated Amount (kg)
Initial biomass composition			
Cellulose	45.5	—	45.5
Hemicellulose *	27.0	—	27.0
Lignin	21.1	—	21.1
Extractives	4.6	—	4.6
Mineral matter/ash	2.2	—	2.2
Component recovery/loss			
Cellulose retained/recovered	—	78% of initial cellulose	35.5
Cellulose loss	—	22% of initial cellulose	10.0
Hemicellulose/pentosan accounted for *	—	≈74% of initial fraction	≈20.0
Pentosan loss *	—	≈26% of initial fraction	≈7.0
Lignin accounted for	—	86% of initial lignin	18.1
Precipitated lignin recovered	—	48.3% of initial lignin	10.2
Lignin loss	—	14% of initial lignin	≈3.0
Other process streams			
Aqueous organics	—	Not fully quantified	NR
Gaseous carbon	—	Not separately quantified	NR
Imported process energy	—	Complete balance not reported	NR
Internally generated energy	—	Not evaluated	NR

Basis: 100 kg dry sugarcane bagasse. NR: Not reported sufficiently for quantitative allocation. * The original study reports the detailed process balance primarily in terms of pentosans. Therefore, values derived from the reported 26% pentosan loss should be regarded as approximate representations of the hemicellulose-related balance rather than a complete molecular hemicellulose balance. Source: Adapted and calculated from Rocha et al.; initial composition cross-checked against Canilha et al. [27,40].

**Table 9:** Multiscale validation framework for fractal-inspired biorefinery design.

Scale	Design Variable	Performance Metric	Validation Method
Material	Pore hierarchy and connectivity	Accessible area, diffusivity, permeability	Imaging, adsorption, diffusion/permeation tests
Catalyst	Active-site and pore distribution	Conversion, selectivity, stability	Reactor experiments
Reactor	Channel/manifold geometry	Pressure drop, mixing, residence-time distribution	CFD and flow experiments
Process	Unit integration and product routing	Energy and carbon efficiency	Process simulation and mass/energy balances
Economic	Equipment and operating requirements	Capital cost, operating cost, profitability	Techno-economic analysis
Environmental	Resources, energy and emissions	Climate change and other environmental impacts	Life-cycle assessment

## Data Availability

No new data were created in this narrative review. Data sharing is not applicable.
